# O Condicionamento Farmacológico pode ser Resgatado pela Dexmedetodina: Como um Sedativo pode Mitigar Lesões de I/R?

**DOI:** 10.36660/abc.20230696

**Published:** 2023-11-14

**Authors:** Leonardo Maciel

**Affiliations:** 1 Instituto de Biofísica Carlos Chagas Filho Universidade Federal do Rio de Janeiro Rio de Janeiro RJ Brasil Instituto de Biofísica Carlos Chagas Filho Universidade Federal do Rio de Janeiro, Rio de Janeiro, RJ – Brasil; 2 Campus Professor Geraldo Cidade Universidade Federal do Rio de Janeiro Duque de Caxias RJ Brasil Campus Professor Geraldo Cidade Universidade Federal do Rio de Janeiro, Duque de Caxias, RJ – Brasil

**Keywords:** Dexmedetomidina, Pharmacology, Mitofagia

O infarto agudo do miocárdio (IAM) é uma condição grave que afeta milhões de pessoas em todo o mundo.^1^ Durante o IAM, o suprimento sanguíneo ao músculo cardíaco é interrompido, reduzindo a disponibilidade de nutrientes e oxigênio. Restaurar o fluxo sanguíneo é essencial para salvar o músculo cardíaco; paradoxalmente, a reperfusão não é um mecanismo inteiramente benigno e induz lesões de reperfusão.^2^ Portanto, tratamentos para atenuar lesões de isquemia-reperfusão (I/R) são necessários. Até o momento, não existem tratamentos farmacológicos totalmente eficazes para aliviar lesões de I/R em pacientes com IAM. No entanto, o condicionamento farmacológico com Dexmedetomidina, medicamento utilizado principalmente como sedativo e analgésico, tem sido relatado como um agente cardioprotetor promissor contra lesões de I/R.

Devido aos seus efeitos sedativos e analgésicos, a Dexmedetomidina é um agonista seletivo do receptor alfa-2 adrenérgico (α2-AR) comumente utilizado em hospitais, especialmente em unidades de terapia intensiva e salas cirúrgicas. Embora sua associação com proteção cardíaca tenha sido sugerida em estudos anteriores,^3 - 7^ o trabalho de Chen et al.,^8^ tema deste minieditorial, aprofundou-se na compreensão do mecanismo subjacente de cardioproteção da Dexmedetomidina. Assim, além de demonstrar que o pré-condicionamento farmacológico com Dexmedetomidina melhora a função cardíaca e reduz a área de infarto do miocárdio após I/R, um dos achados mais significativos deste estudo foi a relação entre Dexmedetomidina e mitofagia.^8^

A mitofagia é um processo fundamental para a manutenção adequada da saúde celular, envolvendo a degradação de mitocôndrias danificadas^9^ e desempenhando um papel crítico na preservação da função cardíaca e na prevenção de lesões de I/R.^10 , 11^ Chen et al. demonstraram que a Dexmedetomidina melhora a manutenção do equilíbrio adequado da mitofagia, suprimindo a degradação excessiva das mitocôndrias funcionais.^8^ Isto resultou em mitocôndrias mais intactas pós-I/R, com manutenção do potencial de membrana mitocondrial e consequentemente redução da produção de espécies reativas de oxigênio. Esses achados são cruciais, uma vez que a manutenção estrutural e funcional das mitocôndrias é essencial para a sobrevivência e funcionamento celular.^2 , 9^ Além disso, se espera que a cardioproteção esteja intimamente envolvida com a homeostase mitocondrial pós-isquêmica.^9^ A Dexmedetomidina também reduziu a formação de autofagossomos, um marcador de mitofagia excessiva. Isto sugere que a Dexmedetomidina poderia modular significativamente a mitofagia, preservar as mitocôndrias funcionais e proteger os cardiomiócitos contra lesões de I/R.

A ativação dos receptores α2-AR parece crucial para os efeitos cardioprotetores da Dexmedetomidina. Quando a ioimbina, um bloqueador do receptor α2-AR, foi administrada com Dexmedetomidina, o efeito cardioprotetor foi revertido. Isto enfatiza a contribuição dos receptores α2-AR para a resposta cardioprotetora contra lesão de I/R. Outros estudos já demonstraram o envolvimento da ativação deste receptor.^3 , 6^ De facto, é relatado que a jusante da ativação deste receptor pode culminar na ativação de PI3K/AKT.^3^ AKT é conhecido por ser um regulador e indutor da mitofagia, mas ainda não se sabe como a mitofagia é regulada com precisão pela AKT para prevenir danos à célula. No entanto, o potencial da membrana mitocondrial parece desempenhar um papel na indução do refinamento da mitofagia. Quando o potencial de membrana mitocondrial é mais positivo, a proteína serina/treonina quinase (Pink1) acumula-se na membrana externa, normalmente danificada, através da formação de um grande complexo com a proteína TOM e sofre autofosforilação intermolecular, levando à sua ativação, resultando na ubiquitinação de proteínas da membrana mitocondrial externa e ativação da autofagia.^12^ Curiosamente, a AKT também pode regular a autofagia mitocondrial seletiva^10^ através de mecanismos que envolvem a manutenção do potencial de membrana mitocondrial^13^ e a regulação de Pink1;^10^ também pela ativação do mTOR, que regula a autofagia e o controle de qualidade mitocondrial.^14^

Em resumo, este estudo fornece evidências promissoras do potencial da Dexmedetomidina como tratamento contra lesão de I/R miocárdica. Ao suprimir a mitofagia excessiva e preservar a função mitocondrial, a Dexmedetomidina pode ser crucial na proteção do coração contra danos resultantes de I/R. No entanto, é essencial reconhecer que este estudo foi realizado num modelo animal, sendo necessárias mais pesquisas para confirmar estes resultados. Os próximos passos deverão evoluir para modelos pré-clínicos utilizando animais de grande porte, como porcos, e depois serem analisados em ensaios clínicos randomizados.
