# Supplementary material for: O Condicionamento Farmacológico pode ser Resgatado pela Dexmedetodina: Como um Sedativo pode Mitigar Lesões de I/R?
Source: Arq Bras Cardiol. 2023 Nov 14;120(10):e20230696. [Article in Portuguese] doi: 10.36660/abc.20230696 (PMC10697677; doi:10.36660/abc.20230696)
Supplement: Translation

# Translation: Pharmacological Conditioning is Salvageable by Dexmedetomidine: How a Sedative Can Mitigate I/R Injuries?

**Authors:** Leonardo Maciel

**Affiliations:** 1 Brazil Instituto de Biofísica Carlos Chagas Filho Universidade Federal do Rio de Janeiro, Rio de Janeiro, RJ – Brazil; 2 Brazil Campus Professor Geraldo Cidade Universidade Federal do Rio de Janeiro, Duque de Caxias, RJ – Brazil

**Keywords:** Dexmedetomidine, Farmacologia, Mitophagy

Acute myocardial infarction (AMI) is a severe condition affecting millions worldwide.^1^During AMI, the blood supply to the heart muscle is interrupted, reducing the availability of nutrients and oxygen. Restoring blood flow is essential to save the heart muscle; paradoxically, reperfusion is not an entirely benign mechanism, and it induces reperfusion injuries.^2^Therefore, treatments to mitigate ischemia-reperfusion (I/R) injuries are necessary. So far, there are no entirely effective pharmacological treatments to relieve I/R injuries in patients under AMI. However, pharmacological conditioning with Dexmedetomidine, a medication primarily used as a sedative and analgesic, has been reported as a promising cardioprotective agent against I/R injuries.

Due to its sedative and analgesic effects, Dexmedetomidine is a selective agonist of the alpha-2 adrenergic receptor (α2-AR) commonly used in hospitals, especially in intensive care units and surgery rooms. Although its association with cardiac protection has been suggested in previous studies,^3 - 7^the work of Chen et al.,^8^the topic of this short editorial, delved into understanding Dexmedetomidine’s underlying mechanism of cardioprotection. Thus, in addition to demonstrating that pharmacological preconditioning with Dexmedetomidine improves cardiac function and reduces the myocardial infarct area after I/R, one of the most significant findings of this study was the relationship between Dexmedetomidine and mitophagy.

Mitophagy is a fundamental process for properly maintaining cellular health, involving the degradation of damaged mitochondria^9^and playing a critical role in preserving cardiac function and preventing I/R injury.^10 , 11^Chen et al. demonstrated that Dexmedetomidine improves the maintenance of the proper balance of mitophagy by suppressing excessive degradation of functional mitochondria.^8^This resulted in more intact mitochondria post-I/R, with the maintenance of mitochondrial membrane potential and consequently reduced production of reactive oxygen species. These findings are crucial once mitochondria’s structural and functional maintenance is essential for cell survival and functioning.^2 , 9^Additionally, cardioprotection is expected to be closely involved with post-ischemic mitochondrial homeostasis.^9^Dexmedetomidine also reduced the formation of autophagosomes, a marker of excessive mitophagy. This suggests that Dexmedetomidine could significantly modulate mitophagy, preserve functional mitochondria, and protect cardiomyocytes against I/R injuries.

The activation of α2-AR receptors appears crucial for Dexmedetomidine’s cardioprotective effects. When yohimbine, an α2-AR receptor blocker, was administered with Dexmedetomidine, the cardioprotective effect was reversed. This emphasizes the contribution of α2-AR receptors to the cardioprotective response against I/R injury. Other studies have already demonstrated the involvement of this receptor activation.^3 , 6^Indeed, it is reported that downstream of the activation of this receptor can culminate in the activation of PI3K/AKT.^3^AKT is known to be a regulator and an inducer of mitophagy, but it remains unknown how mitophagy is finely regulated by AKT to prevent harm to the cell. However, mitochondrial membrane potential appears to play a role in the induction of mitophagy refinement. When the mitochondrial membrane potential is more positive, the serine/threonine kinase protein (Pink1) accumulates on the outer membrane, normally damaged, through the formation of a large complex with the TOM protein and undergoes intermolecular auto-phosphorylation, leading to its activation, resulting in the ubiquitination of outer mitochondrial membrane proteins and activation of autophagy.^12^Interestingly, AKT can also regulate selective mitochondrial autophagy^10^through mechanisms involving the maintenance of mitochondrial membrane potential^13^and the regulation of Pink1 ;^10^also by activating mTOR, which regulates autophagy and mitochondrial quality control.^14^

In summary, this study provides promising evidence of the potential of Dexmedetomidine as a treatment against myocardial I/R injury. By suppressing excessive mitophagy and preserving mitochondrial function, Dexmedetomidine may be crucial in protecting the heart against damage resulting from I/R. However, it is essential to recognize that this study was conducted in an animal model, and further research is needed to confirm these results. The next steps should progress to preclinical models using large animals such as pigs and then be analyzed in randomized clinical trials.
